# Residents’ perspectives: Does the Saudi general surgery residency training program apply CanMEDS competencies and prepare future leaders?

**DOI:** 10.36834/cmej.68488

**Published:** 2020-12-07

**Authors:** Deena Hadedeya, Ghofran Ageely, Nourah Alsaleh, Hajar Aref, Omar Al-Sharqi, Saif Al-Horani, Osman Hamour

**Affiliations:** 1 Tulane University, School of Medicine, New Orleans, USA; 2 Rabigh Medical College, King Abdulaziz University, Jeddah, Saudi Arabia; 3 Al Noor Specialist Hospital, Makkah, Saudi Arabia; 4 International Medical Center, Jeddah, Saudi Arabia; 5 King Abdulaziz University, Jeddah, Saudi Arabia; 6 King Faisal Specialist Hospital, Dammam, Saudi Arabia; 7 King Faisal Specialist Hospital & Research Center, Jeddah, Saudi Arabia

## Abstract

**Background:**

This study investigates leadership skills and Canadian Medical Education Directives for Specialists (CanMEDS) competencies acquisition within the General Surgery Residency Training Program (GSRTP). The Saudi Commission for Health Specialties (SCFHS) incorporates the CanMEDS Competency Framework into its curriculum to prepare the resident for healthcare needs.

**Methods:**

This is a descriptive-analytical study. A questionnaire was used to collect data from 117 General Surgery residents (GS) at seven institutes in Jeddah, Saudi Arabia.

**Results:**

The GS residents reported an acceptable self-perceived level of Clinical Leadership Skills (mean ± standard deviation). The most dominant skill was working with others (1.98 ± 1.03), followed by demonstrating personal qualities (2.07 ± 0.88), the ability to manage services (2.21 ± 1.37), improving services (2.22 ± 1.84) and last, setting directions (2.39 ± 0.95). Regarding the CanMEDS competencies, the respondents showed a generally positive perception with an “agree” level (Mean = 1.83). Of the CanMEDS competency roles, Collaborator ranked first followed by Professional and then Communicator. Leader competency ranked fourth followed by Health Advocate, Medical Expert and last, Scholar.

**Conclusion:**

The GSRTP residents showed satisfactory self-assessed clinical leadership skills and acquirement of the CanMEDS competencies during their training, which will prepare them to lead in the future.

## Abstract (fr)

**Contexte:**

Cette étude se penche sur les compétences en leadership et l’acquisition de compétences CanMEDS (Canadian Medical Education Directives for Specialists - directives canadiennes en formation médicale pour les spécialistes) au sein du GSRTP (programme de formation en résidence en chirurgie générale). La SCFHS (commission saoudienne pour les spécialistes de la santé) intègre le cadre des compétences CanMEDS dans son programme pour préparer les résidents aux besoins en matière de soins de santé.

**Méthodes:**

Il s’agit d’une étude descriptive et analytique. Un questionnaire a été utilisé pour collecter des données auprès de 117 résidents en chirurgie générale dans sept instituts médicaux à Djeddah, Arabie saoudite.

**Résultats:**

Les résidents en chirurgie générale ont rapporté un niveau perçu acceptable de compétences en leadership clinique (moyenne ± écart type) La compétence la plus dominante était le travail avec les autres (1,98 ± 1,03), suivi par la démonstration de qualités personnelles (2,07 ± 0,88), la capacité de gérer les services (2,1 ± 1,37), l’amélioration des services (2,22 ± 1,84) et, finalement, l’établissement des orientations (2,39 ± 0,95). En ce qui a trait aux compétences CanMEDS, les répondants ont montré une perception généralement positive avec un niveau « d’accord » (moyenne = 1,83). En ce qui a trait aux rôles associés aux compétences CanMEDS, celui de collaborateur s’est classé au premier rang, suivi par celui de professionnel et ensuite de communicateur. Le rôle de chef de file s’est classée quatrième en matière de compétence, suivie de défenseur de la santé, d’expert médical et, finalement, d’érudit.

**Conclusion:**

Les résidents du GSRTP ont montré une satisfaction en matière de compétences de leadership clinique autoévaluées et d’acquisition des compétences CanMEDS lors de leur formation, laquelle les préparera à jouer un rôle de premier plan dans l’avenir.

## Introduction

In 1995, the Saudi Commission for Health Specialties (SCFHS) established the General Surgery Residency Training Program (GSRTP) in Saudi Arabia. It is a five-years structured program. By the end of the program, the trainee shall acquire basic surgical knowledge, clinical surgical skills, and the general principles of professionalism. The program aims to graduate competent surgeons with a high level of cognition, psychomotor skills, and affective and attitudinal experience in the different branches of General Surgery so they can competently vie in the international market. Another important aim of the program is to teach trainees the concepts of professionalism and medical ethics, as well as total quality management and cost-conscious practice.

Anecdotal reports show that few surgery programs officially include leadership skills programs in their curriculum. In 2015, the SCFHS integrated and incorporated the CanMEDS Competency Framework into the GSRTP curriculum. The content of the framework was defined and a shorter developed version of the curriculum was then given to the program designing committee for approval. Furthermore, a manual containing the CanMEDS was distributed amongst GSRTP program directors and residents.^1^

The CanMEDS framework was designed by the Royal College of Physicians and Surgeons of Canada (RCPSC) in the 1990s and formally adopted in 1996. It describes the abilities required for physicians to effectively meet the healthcare needs. These are grouped and integrated under seven domains: Medical Expert, Communicator, Collaborator, Leader, Health Advocate, Scholar and Professional.^2^,^3^ Its success depends on strategic implementation plan which includes standards for curriculum design, teaching methodology, assessment, faculty career development, healthy research environment, human development and other resources and a structured outreach and communication program.^4^ Over the years, the RCPSC conducted series of activities , a national ‘CanMEDS Train-the-Trainer’ workshop for each of the 17 medical schools in Canada and many publications on CanMEDS assessment, development and international applicatios.^2^,^5^,^6^ Up until 2005, Being A manager was one of the CanMEDS domains and has since been replaced by leader. As a leader, the physician collaborates with managers to enhance the healthcare system by improving the clinical, academic and administrative responsibilities as well as fostering effective teamwork.^2^

In the UK, the National Health Service (NHS) mentioned that physicians’ leadership competency needs to be actively involved in the process of healthcare system transformation and the General Medical Council (GMC) considers engagement in quality improvement as “very important.”^7^ Physicians who attended their workshop demonstrated more desire and perceived ability to improve healthcare quality and productivity.^8^ To promote leadership, the NHS introduced the Clinical Leadership Competency Framework which is composed of five domains as follows: Demonstrating Personal Qualities, Working with Others, Managing Services, Improving Services and Setting Direction.^7^ The Health Workforce Australia created the LEADS system: (L)eads self, (E)ngages others, (A)chieves outcomes, (D)rives innovation, (S)hapes systems.^9^

## Methods

A questionnaire was distributed among the GS residents in seven training institutions in Jeddah, a city located in western Saudi Arabia. It was designed to elicit their impression of whether the CanMEDS leadership skills and competencies were well explained, properly expressed and eventually implemented in the GSRTP.

The CanMEDS framework competencies were obtained from the official website of the RCPSC of 2015. The points mentioned under the CanMEDS section in the 2015 Saudi GSRTP curriculum were included in the questionnaire. The questionnaire consists of three sections: 1) demographic data, 2) a resident’s clinical leadership competencies**** and 3) the GSRTP competencies based on the CanMEDS. A Likert scale of a five-point ordinal scale was used (Table 1).

**Table 1:** Score of perception statements and their weight ranges.

	Positive Perception		Neutral	Negative Perception	
Questionnaire Statements	Strongly Agree	Agree	Uncertain	Disagree	Strongly Disagree
	1	2	3	4	5
Likert Scale Weight Range	(1 - 1.80)	(1.81 - 2.60)	(2.61 - 3.40)	(3.41 - 4.20)	(4.21 - 5)

Before the final questionnaire was given to the residents, the text and the statement’s clarity were reviewed by a professional academic staff and a statistician. The statements among each variable were analyzed by Pearson Correlation test. Pilot studies were conducted twice with a sample size of 81 respondents and rounded twice for greater reliability. The purpose of the pilot study is to test for comprehensibility and ensure that the respondents will understand the questionnaire. The consistency of the questionnaire was determined using alpha coefficients (Cronbach’s alpha for reliability analysis). In the first round the Alpha Coefficient reliability was (*r* = 0.932) and in the second round (*r* = 0.928), therefore, no modifications were done.

The data collection commenced three weeks after distribution and continued for one week. Program directors were blinded to respondent identities and had no access to the raw data. Ethical and scientific approval was granted by King Faisal Specialist Hospital and Research Center’s Institutional Review Board. The coded data were analyzed using the Statistical Package for Social Science (SPSS) Version 21. A chi-square test was used to find the relationship between independent and dependent variables, whereby p-value = 0.05 was considered significant. For dependent variables, we calculated the mean, standard deviation (SD) and ranked the statements.

## Results

One hundred seventeen GS residents from seven training centers received the questionnaire and consented to read and fill it out. All the training centers apply the same curriculum which is sited by the SCFHS. A total of 95 (81%) questionnaires were received and analyzed and the demographic data are shown in Table 2.

**Table 2:** Demographic data of the 95 Participants in this study.

Demographic Data		Frequency	Percentage
Gender	Male	58	61.1%
	Female	37	38.9%
Age group (in years)	20 – 25	21	22.1%
	26-30	50	52.6%
	31-35	10	10.5%
	36 and over	14	14.7%
Number of years spent in the program	1-3	58	61.1%
	4-5	33	34.7%
	≥ 6	4	4.2%
Total		95	100%

Clinical Leadership Competency Framework was tested with an aim to evaluate residents’ clinical leadership skills, including their ability to work with others, demonstrate personal leadership qualities, manage and improve services as well as the ability to set directions (Table 3).

**Table 3:** Identifying leadership qualities rating their average with standard deviation and the rank each item attains.

Clinical Leadership Competency	Mean	SD	Rank
Working with Others Developing Network Building and Managing Relationships Encouraging Contribution Working with Team	1.98 Agree	1.03	First
Demonstrating personal Qualities Self-Awareness Managing Yourself Personal Development Acting with Integrity	2.07 Agree	0.88	Second
Managing Services Planning Managing Resources Managing People Managing Performance	2.21 Agree	1.37	Third
Improving Services Ensuring Patient Safety Critically Evaluation Encouraging improvement and innovation Facilitating Transformation	2.22 Agree	1.84	Fourth
Setting Directions Identifying the contexts for change Applying Knowledge and Evidence Decision Making Evaluating Impact	2.39 Agree	0.95	Fifth

The third section of the questionnaire assessed each resident’s opinion on acquiring the CanMEDS competencies during their residency training and its relationship with Clinical Leadership. Each competency was examined with four to seven statements. A chi-square test of independence was performed to examine the relationship between each CanMEDS competency as one of the components of the Saudi GSRTP and Clinical Leadership competencies. The results between these variables were significant (*p* < 0.05). The mean, SD, rank and significances value for each competency can be found in Table 4.

**Table 4:** The perception levels among the CanMEDS competencies independent variables.

CanMEDS competencies	Mean	SD	P	Rank	Class
Medical Expert	2.28	0.859	0.000	6^th^	Agree
Communicator	1.92	0.748	0.000	3^rd^	Agree
Collaborator	1.85	0.786	0.000	1^st^	Agree
Leader	2.11	0.848	0.000	4^th^	Agree
Health Advocate	2.15	0.811	0.000	5^th^	Agree
Scholar	2.48	0.902	0.000	7^th^	Agree
Professional	1.88	0.738	0.000	2^nd^	Agree
Total	1.83

In general, the respondents showed a positive perception with a mean “agree” level =1.83. Amongst independent variables, Collaborator ranked first followed by Professional then Communicator. The Leader competency ranked fourth followed by Health Advocate. Interestingly, Medical Expert ranked only 6^th^ followed by Scholar.

## Discussion

In this study, we endeavored to evaluate the impact of incorporating the CanMEDS Competency Framework in the Saudi GSRTP on preparing competent clinicians to be leaders in their practice and fit comfortably into an international workforce. We assessed the GS residents' leadership skills using self-assessments and their acquisition of the CanMEDS Competencies in the Saudi GSRTP. Since the release of the first CanMEDS Competency Framework, many countries adopted it in training and practice.^5^,^10^,^11^ The Framework implication is broadly studied nationally within different specialties which led to several updates and improvement.^12^-^17^ However, there is limited information on international application, particularly in Saudi Arabia.^18^.^19^

For doctors to be merely medical experts is no longer acceptable according to Clark.^3^ Other competencies including appropriate management and leadership skills should be integral elements of medical training, education, practice and medical students’ and doctors’ selection.^3^ Many countries – including the UK, Canada, Denmark and Australia – leadership framework is included as part of the curriculum.^3^,^20^

The Canadian Medical Association (CMA) reported that leadership in medicine is in great need and currently at a void.^21^ In Collins-Nakai (2006), a review of recent focus groups series where the CMA asked its members for their views on leadership and whether there was a need to develop leaders in medicine, he found that physicians felt they are not well-equipped to provide the kind of leadership needed in today's increasingly complex healthcare environment.^21^ They also added that leadership skills were not acquired in their training programs.^21^ In our study, the residents expressed satisfaction with their leadership skills and obtaining CanMEDS Leadership Competency in the GSRTP. However, the Leader Competency ranked only fourth of the seven Competencies. This feedback could indicate that residents need more workshops/courses and/or training in this field.

Surgical trainees among the theater team in South Yorkshire, UK, demonstrated acceptability for the introduction of a workplace-based assessment of nontechnical skills of surgeons including Situation Awareness, Decision Making Communication and Teamwork and Leadership.^22^ On the other hand, trainees may not value all competencies equally.^14^

Many of the residency training program directors believe that teaching the medical expert role is easier and less challenging than teaching the other roles of the framework.^23^ Despite that, it’s application faces many challenges as gap in education, resistance to change, faculty overload and a lack of resources that work against adopting the framework.^4^,^6^ In our study, we found that there is a significant relationship between the Medical Expert domain and Clinical Leadership. Our participants agree that they have acquired the skills to be a Medical Expert including important clinical data collection and interpretation to reach a diagnosis and a decision all with the help of the necessary investigations, as well as up-to-date and evidence-based knowledge and practice.

Discussions of professionalism, teaching of communication skills and collaboration with other healthcare workers and families are now included in the Canadian residency training programs as mandated by the RCPSC.^12^ In this study, the collaborator role ranked first followed by professional and then Communicator. Verma S. *et al* reported in a single institute study on 33 residency programs that little is known about teaching and evaluating the role of the Health Advocate.^24^ Our residents were satisfied with the obtained skills during their residency training as Health Advocate, which ranked fifth.

The physician should remain updated through career development and self-promotion and teaching all health care professionals throughout his career.^2^ Interestingly, the scholar role ranked last among all other competencies which could be a concern.

## Conclusion

This study reveals GSRTP residents’ self-assessed satisfactory Clinical Leadership Skills. They also confirmed and embraced their acquisition of the CanMEDS competencies during their residency program implemented by SCFHS, including leadership competency, which will prepare them for leadership positions in the future. Still, more research is needed to assess objectively the residents’ leadership skills and evaluate deficiencies and difficulties in order to better apply this framework.

Like many other studies, ours has its limitations, primarily being a self-reported study and in a single city. Further research is needed at a wider range by including other cities and to evaluate Program Director’s knowledge and insight about the CanMEDS application in residency training. Also, comparing the Program Directors’ evaluation for the residents with the residents’ self-assessment would not only be enriching and informative but also beneficial to avoid possible bias. As such, we strongly recommend more research to further investigate CanMEDS implementation and detect areas of improvement.
